# Genetic quality: a complex issue for experimental study reproducibility

**DOI:** 10.1007/s11248-022-00314-w

**Published:** 2022-06-25

**Authors:** Atsushi Yoshiki, Gregory Ballard, Ana V. Perez

**Affiliations:** 1 Experimental Animal Division, RIKEN BioResource Research Center, Tsukuba, 3050074 Japan; 2 Comparative Medicine and Quality, The Jackson Laboratory, Bar Harbor, ME 04609 USA; 3 Humodigen, Delmar, NY 12054, USA

**Keywords:** Genetic monitoring, Genetic quality, Genetic modification, Reproducibility

## Abstract

Laboratory animal research involving mice, requires consideration of many factors to be controlled. Genetic quality is one factor that is often overlooked but is essential for the generation of reproducible experimental results. Whether experimental research involves inbred mice, spontaneous mutant, or genetically modified strains, exercising genetic quality through careful breeding, good recordkeeping, and prudent quality control steps such as validation of the presence of mutations and verification of the genetic background, will help ensure that experimental results are accurate and that reference controls are representative for the particular experiment. In this review paper, we will discuss various techniques used for the generation of genetically altered mice, and the different aspects to be considered regarding genetic quality, including inbred strains and substrains used, quality check controls during and after genetic manipulation and breeding. We also provide examples for when to use the different techniques and considerations on genetic quality checks. Further, we emphasize on the importance of establishing an in-house genetic quality program.

## Introduction

During the last 15 years, awareness by the scientific community on the lack of reproducibility from published studies in general, but most importantly from animal studies, has increased. It has been estimated that 36% of the cost of preclinical research is spent on irreproducible experiments due to errors of reagents used and materials including animals (Freedman et al. 2015). Research resources including model organisms such as genetically-modified mouse strains reported in scientific literature often lack critical details so that the study can be reproduced (Percie du Sert et al. 2020). This, coupled with the very low transferability of animal studies to clinical research (Leenaars et al. 2019), are of major concern and needs to be addressed in order to improve drug research and for obvious ethical reasons. Several causes for these have been suggested, such as: lack of statistical power analysis, poor experimental design, health and well-being of animals used, etc. For the purpose of this paper, we will focus on addressing the genetic quality of mice. When working with animal models of disease there are a series of considerations that scientists have in mind in the experimental design like the animal species, weight, sex and age of the animal, health and feed. However, genetic quality is often overlooked albeit being a very important factor. In this review, we will try to address the different aspects of genetic quality that need to be considered when using different inbred strains or genetically modified mice.

## Inbred strains

For the last 100 years, over 450 inbred mouse strains have been described in the literature since the generation of the first inbred strain (Beck et al. 2000). The first inbred mouse strain, DBA, was started in 1909 by Dr. C. C. Little to try to understand the genetics of cancer susceptibility (Paigen 2003). Genetic theory of tumor transplantation was formulated and confirmed experimentally by Drs. C. C. Little and Ernest E. Tyzzer in 1914–1916 (Little and Tyzzer 1916). In 1913 Dr. Halsey Bagg acquired a stock of albino mice to study from a commercial dealer and began breeding a pedigreed line of these mice with the name of Bagg Albino (Potter 1985). The Bagg albino mice later contributed as one of the parents in the development of inbred strains A, CBA, C3H and BALB/c to demonstrate segregation of the cancer incidence among the strains (Potter 1985; Beck et al. 2000). The C57 family of mouse strains such as C57BL, C57L, C57BR, C58 were generated by Dr C. C. Little in 1921 (Festing 1979; Morse 2007). Inbred strains such as C57BL/6, C3H and BALB/c are used widely today as general-purpose standard strains (Festing 1979). They are useful because such genetically-defined mice are stable, breed relatively well, have acceptable background noise, have a convenient short life-span, and are well documented with substantial amount of detailed characterized genetic information (Festing 1979).

According to the definition by the committee on standardized genetic nomenclature for mice in 1952, 20 generations of full-sib mating is the minimum level of inbreeding required for a strain of mice to be designated as “inbred” (Green 1981). At 20 generations, on average at least 98.7% of the loci in each mouse are homozygous. Each inbred strain is also isogenic (genetically identical) because all individuals trace back to a common ancestor in the twentieth or a subsequent generation. F1 hybrids, i.e. the first-generation of crossbred between two inbred strains are also useful in biomedical research because of their isogenicity. The isogenic feature of the inbred strains is particularly important to conduct reproducible experiments with minimal variability if it is carefully controlled.

The inbred C57BL/6 J mouse strain was the second mammalian genome sequenced fully after human (Mouse Genome Sequencing Consortium, 2002; Church et al. 2009). The genomes of twelve classical inbred mouse strains and four wild-derived strains have also been sequenced and sequence data has become available from the public repository (Lilue et al. 2018). Because of the enriched genome information, inbred mouse strains have been used as the premier organism for modeling human disease and functional genomic studies today.

## Congenic mice and the importance of substrains

The reproducibility that inbred strains bring to research is also important when dealing with genetically modified mice. When generating a genetically modified strain through the use of pronuclear injection (Isola and Gordon 1991; Ittner and Götz 2007), homologous recombination (DeChiara et al. 1990; Babinet and Cohen-Tannoudji 2001) or genome editing (Yang et al. 2014), it is ideal to use a well characterized inbred mouse strain, as this allows for an easier phenotypic characterization of the newly introduced mutation. Which strain, however, can be an important consideration as the background strain the mutation is on, can significantly affect the phenotype of the mutation. One classic example of this is the difference in phenotype of the *Lep*^*ob*^ and *Lepr*^*db*^ mutations on C57BL/6 vs C57BLKS/J mice (Coleman 1978). Both strains develop obesity and diabetes with these mutations, but whereas the diabetes is transient in the C57BL/6 mice, it is lifelong and much more severe in the C57BLKS/J mice. Threadgill et al. (1995), also showed that the knockout of the epidermal growth factor receptor in three different strains was lethal in all three strains, but was embryonic lethal on the CF-1 and 129 genetic backgrounds while the same mutation on a CD-1 background survived until 3 weeks after birth. There are many more examples of how mutations show a different phenotype depending on the genetic background or more practically, depending on the inbred strain or more precisely, substrain used.

With the advent of CRISPR/Cas 9 genome editing technology, it is easier to generate a mutation or introduce several mutations simultaneously, directly in the desired genetic inbred strain. However, there are still times in which a mutation might need to be moved to a different genetic background strain. Also, if an experiment requires the cross of two or more genetically modified mutants in different inbred strains, the resulting mice are typically backcrossed to the same inbred strain to generate a congenic strain (Rogner and Avner 2003). For example, if a targeted mutation was generated in 129 mice, it is often advantageous to then transfer the mutation to another strain through backcrossing. To do this, the original 129 mice, called the donor strain, which contains the targeted mutation, are bred to a recipient strain, like C57BL/6J or C57BL/6N, over multiple generations. Each generation is tested to ensure the targeted mutation is still present. After 10 generations of backcrossing, the mice still have the mutation of interest, but now shares over 99% of identity with the recipient strain (Lutz et al. 2012). There are several reasons for why this might need to be done. For example, certain inbred strains are commonly used in different types of research. The most common background strain for genetically altered mice is C57BL/6, which is a commonly used general purpose laboratory strain, however, we will see below the importance of substrains. BALB/c substrains are widely used, particularly in studies of immunology. C3H substrains are used in areas like cancer, infectious disease and cardiovascular research, and DBA substrains are used in cardiovascular and glaucoma research (Lutz et al. 2012). In order to directly compare results from a novel mutation to the existing literature, it may therefore be necessary to move the mutation to a different strain. Also, it is possible that the recipient strain might be a more desirable model for the mutation, perhaps because it has a more pronounced phenotype. In terms of genetic integrity, this backcross process also removes any off-target mutations which may have occurred during the mutagenesis process, as long as these unwanted mutations are not in the immediate area surrounding the mutation of interest, as the rest of the genome in congenic mice comes from the recipient strain. This process can be expedited using genetic markers, such as, Single Nucleotide Polymorphisms (SNPs), used for genetic monitoring, using a process called marker assisted speed congenics (Wakeland et al. 1997). The use of SNPs, and other markers, for genetic monitoring is discussed in more detail below.

One potential pitfall for congenic mice, and in fact for all inbred mice, is ignoring the differences among different substrains within a given strain. For example, erroneously assuming that all C57BL/6 mice, are identical. This is not true. A recent review by Mekada and Yoshki (2021) gives an overview of the many phenotypic differences seen in the different C57BL/6 (B6) substrains, and the most common C57BL/6 substrains are C57BL/6J and C57BL/6N. However, there are many commercial distributors which have their own C57BL/6 substrain. C57BL/6 J and C57BL/6NJ are produced by the Jackson Laboratory, C57BL/6JBomTac and C57BL/6NTac are produced by Taconic Biosciences, C57BL/6JOlaHsd and C57BL/6NHsd are produced by Envigo, etc.

C57BL/6J and C57BL/6NJ, while originally from the same inbred strain, have been bred separately since 1951, and have acquired multiple genetic differences (mutations or polymorphisms), independently of one another. Simon et al. (2013) compared C57BL/6J and C57BL/6NJ SNPs and indels and found 34 SNPs and 2 indels in coding regions which were different between the two substrains. These included a mutation which led to blindness in C57BL/6NJ mice, *Crb1*^rd8^, and a nucleotide deletion in the nicotinamide nucleotide transhydrogenase gene which causes the loss of exons 7–11 in C57BL/6J mice (Freeman et al. 2006). It is imperative to be aware of such differences and it is important to emphasize that, for example, C57BL/6J and C57BL/6NJ cannot be used interchangeably. Bourdi et al. (2011) found completely different results when they studied liver injury in C57BL/6NJ c-Jun N-terminal kinase 2 (JNK2) knockout mice when they used C57BL/6J mice as controls vs C57BL/6NJ mice. They found that if they used C57BL/6J in their control experiment it looked like the JNK2 knockout mice had increased liver damage. In actuality, when the C57BL/6NJ JNK2 knockout mice were compared to the appropriate control, C57BL/6NJ mice, the JNK2 mutation was actually protective, leading to lower ALT levels, a biomarker for liver damage, in the blood. The difference in results was because C57BL/6NJ mice are more susceptible to liver damage than C57BL/6J mice with the agents tested (Acetaminophen and Concanavalin A). This is an important example of why not just strain, but also substrain, matters.

## Genetically modified strains

**Fig. 1:**
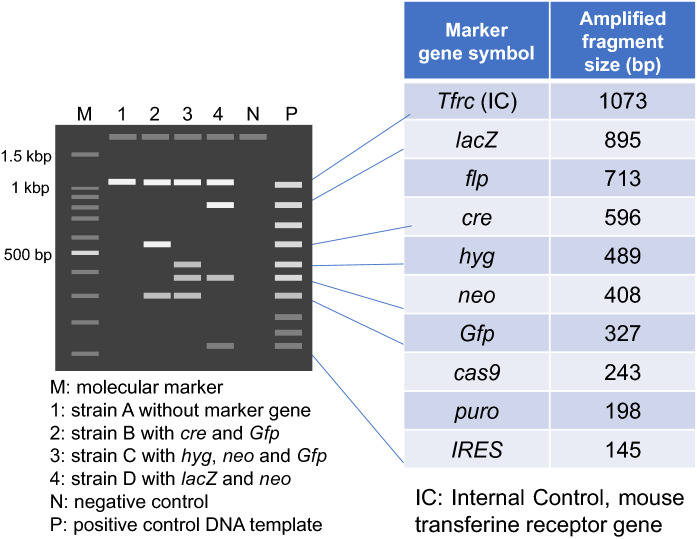
Schematic image of agarose gel of Multiplex PCR test result to detect marker genes widely used in genetically altered mice. The* Tfrc *gene as internal control was commonly detected in all strains A-D

**Fig. 2:**
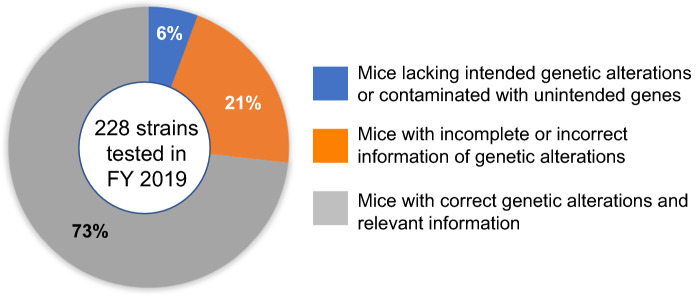
Genetic quality of mouse strains in Japan

As the mouse is a living organism, genetic drift, spontaneous mutations, and inadvertent cross-contamination with other strains, can occur during breeding and should be minimized for reproducible experiments. In addition, many of the “tools” or techniques used to introduce genetic modifications bring potential problems if they are not considered or verified. It has become increasingly difficult for scientists that use genetically modified mouse models to analyze and interpret experimental results appropriately due to oversight of the different risks these techniques may introduce if left unchecked. It is strongly recommended to confirm the genetic quality of genetically modified mouse models before starting a full-scale experiment. As a typical example, genetic contamination tests by using primers to amplify widely used marker genes such as: *neo*, *Pgk-neo*, *Tk-neo*, *IRES*, *lacZ*, *GFP*, *Cre*, *Flp*, *Puro*, and *Hyg*, among others (Fig. 1; Nakata et al. 2009), on over 200 mouse strains submitted to RIKEN BRC in 2019, found that 21% were associated with incorrect information of the genetic modification and 6% contained actual genetic contamination (Fig. 2). It was also reported that genetic tests of approximately 400 mutant mouse lines submitted to The Jackson Laboratory and the Mouse Mutant Resource and Research Centers detected 15% of unintended mutations or genetic markers (Lloyd et al. 2015).

## Strains generated by Pronuclear Microinjections

This technique was the first one used to introduce genetic modifications to the mouse genome (Palmiter and Brinster 1985). Transgenic mice are generated by pronuclear microinjection of a one cell fertilized egg with a hybrid gene, usually consisting of the regulatory promoter of a gene fused to the structural elements of another gene (Palmiter and Brinster 1985; Polites and Pinkert 1994; Kim et al. 1990). Larger bacterial artificial chromosomes (BACs), P-1 derived artificial chromosome (PAC), and yeast artificial chromosome (YAC) DNAs containing the coding gene, intronic and regulatory regions necessary for precise spatiotemporal gene expression have also been used for microinjection to generate transgenic mice for functional genomic studies (Roguev and Krogan 2008; Yang et al. 1997; Duff et al. 2000; Lamb et al. 1999). Over 7,000 transgenic mouse lines have been registered and available in the International Mouse Strain Resource database (Eppig et al. 2015).

The expression of the fusion transgene is primarily regulated by the promoter, which can either act ubiquitously in all cell-types or in a cell-type specific manner (Palmiter and Brinster 1986; Kim et al. 1990). Together with the promoter, the integration site, and copy number of the transgene, can influence expression levels of the transgene (Tinkle et al. 1994). Microinjected DNA fragments are integrated randomly in the genome usually as a tandem array of multiple copies. The random integration of the same DNA construct in different founder lines can result in multiple lineages with varying expression patterns, depending on the number of copies and integration sites of the transgene in the genome (Tinkle et al. 1994).

**Fig. 3:**
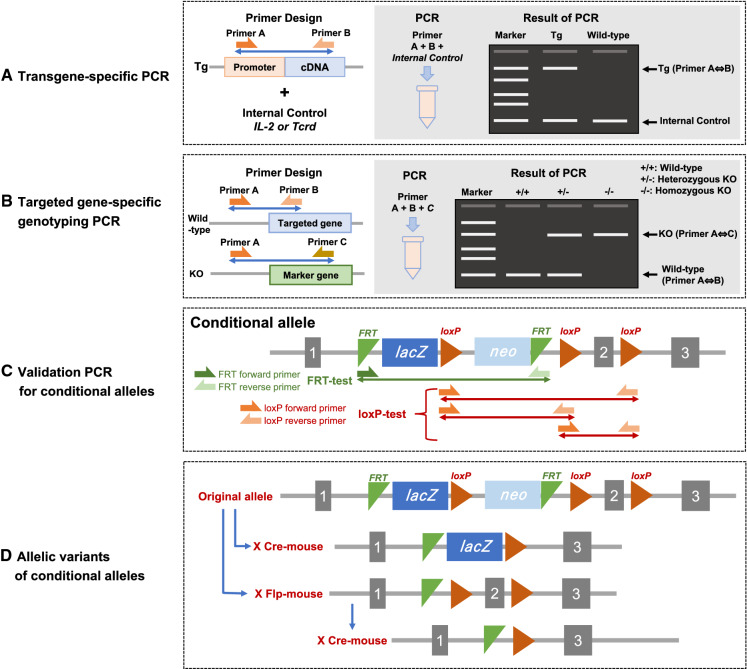
PCR tests for genetically altered mouse strains

The quality of the transgenic mice can be evaluated by the presence of the transgene, intactness of the entire transgenic construct being introduced, copy number, number of integration sites, and the orientation of tandem copies. Accurate information of the transgene integrated on the genome is critical to design valid quality control tests. Furthermore, if possible, sequencing of the transgene amplicon is recommended to verify that it is intact and there have been no rearrangements of the transgene during its concatemerization/recombination, which happens as part of the genome insertion process. There is a recent review that discusses the potential recombination mechanisms that occurs (Smirnov and Battulin 2021). It is recommended that original founder transgenic mice created by pronuclear microinjection are identified by Southern blot analysis. Southern blot analysis provides key information of the structure, integrity, and sometimes copy number of the inserted transgene (Nagy et al. 2003). Based on such former reliable information about the inserted transgene, transgene-specific PCR tests can be designed by using a promoter-specific forward primer and a structural sequence-specific reverse primer to detect transgenic carrier mice with an appropriate positive and negative controls (Fig. 3A). If there are several similar reporter transgenic lines, in a mouse breeding facility, containing fluorescent protein variants such as EGFP, CFP, YFP and Venus (Cormack et al. 1996; Tsien 1998; Yang et al. 1998; Nagai et al. 2002) driven by the same promoter or, if the same fluorescent protein gene is driven by different promoters, highly specific PCR primers should be carefully designed in order to distinguish each promoter and reporter genes to check against accidental genetic contamination through breeding. Table 1 summarizes several genetically modifying techniques and their use.

**Table 1:** Genetic modification techniques used

	Pronuclear Injection	CRISPR/Cas9 Fertilized Eggs PI/EP	CRISPR/Cas9 Embryonic Stem (ES) Cells	Embryonic Stem (ES) cells	Breeding genetically altered mice
Use	To study the effect/function or expression pattern of the transgene	Knockout to study the function of endogenous genes Knockin for targeted transgenesis	Knockout to study the function of endogenous genes Knockin for targeted transgenesis	Knockout to study the function of endogenous genes Knockin for targeted transgenesis	To generate multigenic disease models
Method of introduction	Pronuclear Injection of fertilized egg	Cytoplasmic Injection, Pronuclear Injection or Electroporation of CRISPR/Cas9 + oligo DNA or targeting vector	Electroporation of CRISPR/Cas9 + oligo DNA or targeting vector	Electroporation of complex targeting vector and selection of homologous recombinant	Cross breeding
Resulting Modification	Random integration of Transgene	Indel, point mutations, integration of complex allele	Indel, point mutations, integration of complex allele	Indel, point mutations, integration of complex allele	Dependent on parental strains
Gene expression	Dependent on regulatory sequence, copy number, integration site of the Transgene	Dependent of endogenous gene regulation Efficient and stable gene expression in safe habor loci	Dependent of endogenous gene regulation Efficient and stable gene expression in safe habor loci	Dependent of endogenous gene regulation Efficient and stable gene expression in safe habor loci	Dependent on parental strains
Timeline From procedure execution to Fo birth	2–3 months	6 months	Multiple steps/1–2 years	1–2 years	2–3 years to crossbreed multiple strains
Notes	Need to select a stable line from multiple founder lines	Founders are likely to be mosaic. Need to confirm germ-line transmission *EP = electroporation	Need germ-cell competent ES cells, chimera formation and confirm germ-line transmission	Need germ-cell competent ES cells, chimera formation and confirm germ-line transmission	Genetic background depends on parental strains

## Strains generated by Mouse Embryonic Stem Cells

Mice generated by homologous recombination of DNA sequence electroporated into mouse embryonic stem (ES) cells (Evans & Kaufman 1981; Thomas & Capecchi 1987), was the first genetic manipulation technique that allowed targeted modifications of genomic DNA to generate sophisticated mouse models of human disease (Smithies et al. 1985; Doetschman et al. 1987; Mansour et al. 1988; Colledge et al. 1995), and it won the Nobel Prize for the inventors (Vogel 2007). This technique refined the generation of DNA constructs introduced and allowed generation of conditional models (Nagy 2000). The null-knockout, conditional knockout and knockin mouse strains generated by targeted mutagenesis in ES cells increased dramatically during the early 2000s and covered modification of more than half of the coding genome in mice. Since homologous recombination in mice is very inefficient and this is the process used to modify mouse embryonic stem cells, the primary genetic quality test should be a targeted gene-specific genotyping PCR to verify the precise location of the gene insertion and the integrity of the insert itself (Fig. 3B). These targeted ES cells and mouse strain resources are available in high quality from major mouse repositories such as KOMP, MMRRC, EMMA, The Jackson Laboratory and RIKEN BRC (Birling et al. 2021). While early ES cell lines were primarily derived from the 129 strains, ES cell lines with high germline transmission rates are now available for C57BL/6J or C57BL/6N substrains (Pettitt et al. 2009; Tanimoto et al. 2008; Hansen et al. 2008; Zevnik et al. 2014) and some other inbred strains (Bouabe and Okkenhaug 2013). Chimeras of 129 ES cells and C57BL/6 host blastocysts are traditionally crossed with the pertinent C57BL/6 substrain due to the poor breeding performance of the 129 strains. Chimeras formed by using C57BL/6 ES cells can be crossed with the same inbred strains as ES cells to establish inbred knockout mice (Nagy et al. 2003) (Table 2).

**Table 2:**
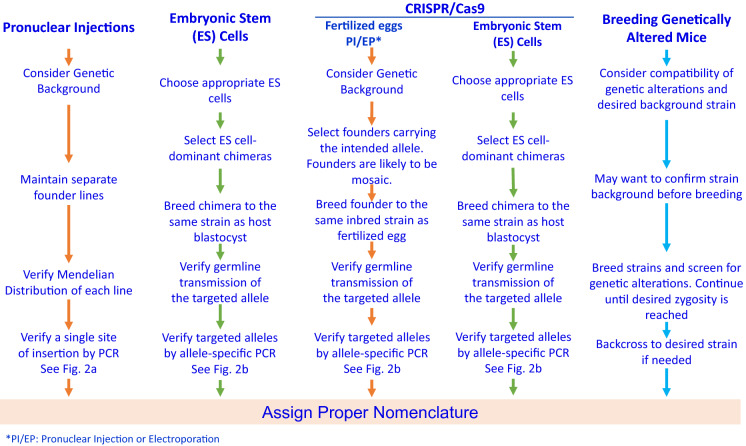
Genetic quality guide chart

## Confirmation of structure of conditional alleles which carry *loxP* or *FRT*

More than 10,000 unique protein coding genes have been mutated in mice using targeted mutagenesis with embryonic stem cells for the last thirty years by the research community (Birling et al. 2021). Conditional mouse models generated by homologous recombination usually incorporate 34-bp sequences that are recognized by specific DNA recombinases (Nagy 2000), and the most widely used are l*oxP* (Sauer and Henderson 1988) and *FRT* (Sandowski 1995) among many other variants (Nagy et al. 2003). For conditional alleles, there are several complex allele structures containing multiple l*oxPs*, *FRTs* and other functional components (Birling et al. 2021; Skarnes et al. 2011). The primary genetic quality test should be the validation of the presence or absence of the conditional alleles, namely *loxP*s and *FRT*s that are necessary for the conditional potential (Fig. 3C) (Nakata et al. 2021). These complex alleles are further modified by crossing Cre or Flp recombinase-carrying mice to generate a null, null reporter or a conditional mouse line (Skarnes et al. 2011). The PCR test should be designed to clearly distinguish genotypes of these allelic variants (Fig. 3D).

## Tet transgenic mice

**Fig. 4:**
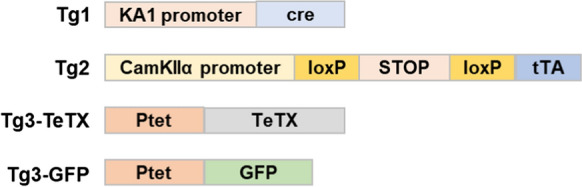
The first triple transgenic line of Tg1 x Tg2 x Tg3-TETX was generated to demonstrate that the action of tetanus toxin (TeTX) blocks neurotransmission in specific neurons in the hippocampus when Dox is not administered. Administration of Dox can reversibly release this blockade of neurotransmission. The second crossbred of Tg1 x Tg2 x Tg3-GFP was to visualize neurons with GFP in the dentate gyrus (DG) of the hippocampus and the pyramidal neurons of CA3, and the expression of GFP disappeared by administration of Dox

Genetic modification technology is essential to analyze experimentally the function of genes in vivo. However, transgenesis and targeted mutagenesis as above do not necessarily provide precise control of gene expression in a site-specific and timely manner. The TET system provides the reversible modifications of the transcriptome, via the action of tetracycline-controlled transcription factors. More than 500 Tet mouse lines have been published for modeling human diseases and analyses of brain functions (Schönig et al. 2013). There are two types of TET systems, Tet-Off and Tet-On expression control system. The gene expression can be turned on/off by introducing a pair of genes, Tet transactivator and tTA/rtTA responsive gene into transgenic mice and adding/removing doxycycline to the drinking water or by intraperitoneal injection, and the gene expression level can also be controlled by the concentration of doxycycline. Therefore, the TET system is used by crossing at least two independent transgenic lines and often combined cre/loxP system as shown in Fig. 4 (Nakashiba et al. 2008). Usually, these mouse mutants are generated by pronuclear injections or homologous recombination, therefore, the genetic quality checks previously described apply here as well.

## Strains generated by genome editing

The advancement of genome editing technologies has made it possible to directly generate various types of genetic modifications by microinjection of genome editing reagents into fertilized eggs in mice and rats without the need to use embryonic stem cells (Wang et al. 2013; Meek et al. 2017), including gene knockout, knock-in, and conditional knockouts. Among the three major genome editing technologies used are zinc-finger nucleases, transcription activator-like effector nucleases (TALENs), and clustered regularly interspaced short palindromic repeats (CRISPR)-associated Cas9 nuclease (CRISPR/Cas9) (Cong et al. 2013). CRISPR/Cas9 is currently the technology most widely used for generation of genetically modified mice. This discovery brought the second Nobel Prize to the field (Cohen 2020).

The CRISPR/Cas9 system consists of the Cas9 nuclease and a single guide RNA (sgRNA) containing the target complementary crRNA and the trans-activating crRNA elements. Since target recognition is mediated entirely by the sgRNA, CRISPR/Cas9 is considered as the most flexible and user-friendly platform for genome editing (Cong et al. 2013). However, recent investigations on the genetic quality of genome edited mice, namely of the founder generation (F0) have uncovered a wider range of on-target genomic changes such as inversions, duplications, structural variants, and complex rearrangements as well as indels and substitutions, including mosaicism and unwanted off-target events (Kraft et al. 2015; Boroviak et al. 2017; Kosicki et al. 2018). Such unintended genomic changes should have occurred in double-strand break repair pathways regulated by host early embryonic cells of F0 animals that have undergone genome editing (Yeh et al. 2019). Therefore, when designing experiments using CRISPR/Cas9, much care should be used in designing the specificity of the sgRNA by using available software that helps zero-in specificity minimizing off target effects, using high fidelity nucleases, and fine-tuning lab protocols. Several publications (Iyer et al. 2018; Dong et al. 2019) have focused on off-target analysis of CRISPR/Cas9 genetically modified mice and it appears to be a consensus that for the most part off-target effects are minimal provided that precautions stated above were taken.

In most cases, the F0 mice are mosaic of heterogenous cells with multiple alleles generated by genome editing (Mizuno et al. 2014). Therefore, appropriate F0 mice with an intended mutation should be screened and carefully bred to establish a new mutant line. Ideally, potential off-target sites should be verified as intact through sequencing. The most advanced genetic quality tests for genome-edited mice include multiplex long-read sequencing and machine learning (Kuno et al. 2022). Once a line with the intended mutation is isolated, the same quality control checks used for the targeted mutation via embryonic stem cells should be used for the endonuclease-mediated mutation in the successive offspring. Additionally, if encountered with unexpected genetic quality control (QC) test results, one should try sequencing whenever necessary.

## Strains generated by targeted transgenesis

Several of the techniques discussed above have been used to target specific, characterized loci in the genome, thus the name Targeted Transgenesis. Targeting a predetermined site in the genome assures efficient and stable expression of a DNA construct, avoiding the significant QC issues associated with random transgenesis (Ohtsuka et al. 2015). Safe harbor loci such as *Rosa26*, *Hprt1*, and *Cd6* gene loci, as well as *Tigre* and *Hipp11* intergenic regions have been used in the mouse to insert CAG promoter driven transgenes, reporter genes, Cre-reporters, *lacZ* genes, human genes, TET system, BAC constructs, etc. (Soriano 1999; Vivian 1999; Heaney 2004; Zeng et al. 2008; Palais et al. 2009; Tasic et al. 2011; Ichise et al. 2014; Madisen 2015; Ma et al. 2017; Browning et al. 2020). Targeted transgenesis can be achieved via conventional ES cell-techniques or microinjection of zygotes with CRISPR/Cas9 reagents and validated by relevant genetic quality control tests according to the genetic modifying techniques used (Table 1).

## Use of standardized nomenclature as a tool of genetic quality

Mouse strain nomenclature is a critical part of animal identification and genetic quality. It follows nomenclature guidelines, including description of all genetic modifications and the inbred strain used with laboratory code assigned by the Institute of Laboratory Animal Research (Eppig 2006). These are often omitted in scientific publications. Recently, the Resource Identification Initiative (Vasilevsky et al. 2013; Bandrowski et al. 2015), has been launched to improve research resource transparency within the biomedical community by promoting the use of unique Research Resource Identifiers (RRIDs). Scientists are encouraged to cite specific RRIDs with the proper strain nomenclature. These concerted efforts will improve the ability to identify exact research resources used in publications and ensure study reproducibility.

## Crossing multiple genetically modified mice

While mice with a single genetic modification are powerful models for scientific research, some research questions can only be addressed by combining multiple genetic modifications onto the same mouse strain. For instance, combining a transgenic line with a knockout line. Attempting to cross two genetically modified mice has its own set of considerations. First, it is essential that the two genetic modifications can independently assort. Ideally, they should be on different chromosomes, though if they are sufficiently spaced on the same chromosome the genes can independently assort, based on the number of centimorgans between them, with 1 cM being a 1% chance for crossing over to occur between the two genes. Complicating this assessment is the fact that, the incorporation site of many transgenes is not known, making it difficult to tell how compatible the transgene may be with a given knockout (Cain-Hom et al. 2017).

While certain modifications may not be compatible because of their relative locations in the genome, they also may be incompatible or difficult for other reasons. Some combinations of modifications may lead to infertility or embryonic lethality. These modifications may be more difficult or impossible to combine. For infertility, if one gene is recessive, then the mice may be able to be maintained as heterozygous, with homozygous mice used in the study, but not as breeders. Alternatively, sometimes reproductive techniques like ovarian transplant and/or artificial insemination may also be used. For mutations that prevent the viability of embryos, sometimes this can be overcome using conditional expression. Systems like Cre, Flp or Tet, which were discussed earlier, can control the expression of a gene either temporally or in specific cells or tissue type (Becher and Waisman 2018). This may enable expression of the required gene either early on to overcome the embryonic lethality, followed by suppression later in the mouse’s life, or if the tissue or cell type of interest is not essential for embryonic development, by conditionally knocking out the gene in that specific tissue, while leaving it fully functional in other tissues or cell types.

In addition, when crossing mice with different genetic modifications, each modification will have its’ own passenger segment. The passenger segment is the portion of the donor strains DNA flanking the modification site. This flanking DNA is always carried with the mutation, even after many rounds of backcrossing, because of the low probability of close portions of DNA independently assorting due to crossing over. While these sections of donor DNA can be minimized by backcrossing, they cannot be eliminated, and are likely to contain at least one functioning gene from the donor strain (Ackert-Bicknell and Rosen 2016). If a passenger gene is important to the study, it may make be difficult to compare these mice to control mice, as it would not be entirely clear if the differences are due to the mutation alone, or also because of differences in the passenger gene between the donor strain and recipient strain (Eisner-Dorman et al. 2009). Table 2 summarizes the different genetic modifications and its appropriate genetic quality checks.

## Genetic monitoring during breeding

The basis of any good genetic monitoring program is consistent husbandry procedures, good recordkeeping, a well-trained staff and a robust testing regime. Examples of procedures which can help prevent any mix-ups within an animal room include trying to separate mice with the same coat color as much as possible, using color coded cage cards, with different colors used for adjacent strains and separate mice with similar strain names like BALB/cJ and BALB/cByJ (Strobel et al. 2015). Having robust husbandry procedures which help mouse room staff avoid mistakes is an essential safeguard against genetic contamination.

Another important feature of a genetic monitoring program is good recordkeeping. Including keeping detailed records of mouse breeding and any mouse transfers. It is essential that all records include proper nomenclature for genetically modified animals to avoid any mix ups. These detailed records can both help avoid contamination of strains, and also aid in tracking down and eliminating contaminated animals, if an instance of genetic contamination does occur.

An essential element to any genetic monitoring program is a well-trained staff. As the staff interact with the mice daily, they are in the best position to notice any changes, either in behavior, breeding or physiology. Particularly with small colonies of mice, noticing deviations and eliminating them from the colony quickly can potentially prevent mutations from becoming fixed within the colony (Fahey et al. 2013). Also, it is the mouse room staff that will carry out all the husbandry procedures and record keeping, so ensuring that procedures are executed accurately, and that all staff are aware of the importance of maintaining genetic integrity in the mice.

While strong husbandry procedures, recordkeeping and a well-trained staff can prevent the vast majority of genetic contaminations, it is still essential to monitor mice for genetic contaminations. Any method that is used to test for accidental genetic contamination must be sufficiently polymorphic so that it can distinguish between all strains in the facility (Fox et al. 2007). Ideally it should also be inexpensive and easy to perform, so that testing can be frequent. The most commonly used markers which meet these criteria are single nucleotide polymorphisms and microsatellites, both of which will be described in more depth in a later section.

While modern molecular techniques for genetic contamination testing are indeed powerful, it is not meant to detect genetic drift. Genetic drift is a phenomenon where spontaneously occurring mutations can become fixed in an inbred colony. Genetic drift accounts for some of the well documented differences between substrains, like lower endotoxin sensitivity in C3H/HeJ vs C3H/HeOuJ (Poltorak et al. 1998), or retinal degeneration in C57BL/6NJ but not C57BL/6J mice (Mattapallil et al. 2012). Genetic drift is especially problematic in small colonies, where unwanted mutations can become rapidly fixed in the strain.

To minimize genetic drift, it is recommended to periodically refresh the breeding stock from a commercial supplier typically at least every 10 generations (Flurkey 2009). Doing so prevents the establishment of a divergent substrain in the colony. Many commercial breeders periodically refresh their breeders from a stock of frozen embryos, greatly reducing genetic drift. Cryopreservation is also recommended for any unique or genetically modified strains both to guard against loss of the colony either from a disaster or genetic contamination, and to prevent genetic drift by in house breeding. Such strains should be cryopreserved, either as embryos or sperm, and rederived every 10 generations to avoid genetic drift.

Genetic monitoring is an essential part of maintaining animal colonies. While the importance of attentive animal care workers and robust husbandry procedures and documentation in preventing strain contamination cannot be overstated, a genetic monitoring program, particularly in facilities which house multiple mice with the same coat color, is an important verification step to catch any incidents of genetic contamination.

## Genetic background testing: SNPs and microsatellites

Before molecular techniques became readily available, genetic background testing was carried out using phenotypic markers (like coat color, fecundity) as well as biochemical tests (like isoenzymes) and immunological tests, like skin grafts (Mahler and Nicklas 2012). The advent of widespread molecular methods for genetic monitoring has allowed much more high-throughput and higher confidence methods for screening for genetic background contamination. The two primary used methods of testing for genetic background involve either microsatellites or SNPs.

Microsatellites, sometimes also called Simple Sequence Length Polymorphisms (SSLPs), are abundant sequences throughout the genome which are made of short tandem repeats (a repeated sequence of 2–6 bases), which are repeated between a few to several dozen times (Grover and Sharma 2014; Benavides et al. 2020). The length of these repeats often differs between different inbred strains. This is because errors in replication of microsatellite regions are relatively common, causing an increase or decrease in the number of repeats present at that site (Dallas 1992). PCR primers, which immediately flank the location of the tandem repeats, can be used to amplify the repeated sequence and verifying its size. By looking at the length of numerous sites throughout the genome, microsatellites can be used in mice (Basta et al. 2004) and other rodents (Bryda and Riley 2008) to easily distinguish between inbred strains.

The other common type of marker used for genetic monitoring are SNPs. SNPs are polymorphic sites which are abundant throughout the genome, are present in both coding and non-coding DNA and typically have only two alleles (Benavides et al. 2020). The location of SNPs, are well documented for many inbred mouse strains, therefore, a panel which distinguishes between common inbred strains can be readily designed with multiple markers over all chromosomes. Information about the SNPs present in different inbred mice strains are readily available from sources including at the Mouse Genome Informatics website (MGI; http://www.informatics.jax.org/home/strain, 2020) and the Mouse Phenome Database (MPD https://phenome.jax.org/genotypes, 2020). There are also published panels of SNPs, such as the one in Petkov et al. (2004), which have markers on all chromosomes and can be used to distinguish between many inbred mouse strains. Additionally, if SNP information is not available for a particular inbred strain, SNP microarrays are a cost-effective way to determine which SNP alleles are present in the order of tens or hundreds of thousands SNP sites (Morgan et al. 2016). SNPs are typically dimorphic, therefore detection methods for screening typically involve either allele specific primers or the use of allele specific probes to distinguish between alleles. One common system used for high throughput SNP genotyping is Kompetative Allele Specific PCR (KASP), which uses allele specific primers, combined with universal energy-transfer (ET) labeled primers (Myakishev 2001). As it uses universal ET primers, the KASP system tends to be less expensive than the other primary method of SNP detection, Real-Time PCR Taqman assay. Taqman assays use site specific primers combined with allele specific probes to determine which SNP allele is present at a given site. Both KASP and Taqman assays are highly sensitive and can be automated for high throughput testing.

A recent publication that will help with genetic quality control testing is the new version of the Mouse Universal Genotyping Array (MUGA) that was originally published in 2015, the MiniMUGA Genotyping Array (Sigmon et al. 2020). It is an Illumina Infinium array-based platform that tests for 11,000 probes at once and it is able to discriminate between mouse substrains from multiple commercial vendors, detection of genetic elements used in DNA constructs when making genetically modified mice and chromosomal sex determination. This with the additional testing we have described in this paper offers a good option for maintaining genetic quality control in the characterization and maintenance of breeding mouse colonies.

Whether using microsatellites or SNPs to monitor for genetic contamination, it is important to test enough markers to distinguish between all the strains and, ideally, substrains present in your facility. Genetic monitoring is of little use if it is not sensitive enough to discover a genetic contamination. SNP or Microsatellite Panels should contain critical markers on each chromosome that are able to distinguish between a large number of inbred strains, including substrains. It is understood that cost is also a factor and therefore larger panels, while more robust in terms of detecting contamination, also tend to be more expensive to run. A smaller panel, which can be run on more mice, with greater frequency, may be preferable. If your facility has many C57BL/6 mice of multiple substrains or unknown substrains, you may want to consider adding markers that distinguish between C57BL/6 substrains. There have been several publications of SNP markers which could be used for that purpose (Mekada et al. 2015; Simon et al. 2013). Whatever the number of markers, verifying the inbred background of strains and substrains, in conjunction with routine verification on the presence or absence of mutations in genetically modified mice, are important components of a thorough genetic quality program.

## International council for laboratory animal science (ICLAS) and genetic quality

The ICLAS laboratory Animal Quality Network Committee has as its goal to raise awareness of the scientific community on the importance of high-quality laboratory animals used for research, as well as to maintain and improve the health and genetic quality on animals used in research (Turner et al. 2015). The Genetic Quality Monitoring Program promotes the development of self-assessment colony monitoring for research facilities and suppliers of rodent breeding, providing guidance and advice on genetic quality testing. In 2016, the GENRef program was established and it provides reference of genomic DNA from the 12 most common inbred/sub-strains of laboratory mice, namely: C57BL/6NTac, BALB/cAnNTac, C3H/HeNTac, 129S6/SvEvTac, C57BL/6J (reg. #664), BALB/cJ (reg. #651), NOD/LtJ (reg #1976), A/J (reg. #646), DBA/2JJcl, C3H/HeJJcl, DBA/2NJcl, FVB/NJcl. The DNA was prepared in batch by the Donor providers (The Jackson Laboratory, Taconic Biosciences and Central Institute for Experimental Animals), and tested for verification by PCR and SNPs.

These samples are available so that research institutions throughout the world could use them to compare the genetic background of their mice to established strains widely used from commercial providers. Research institutes wishing to participate in the program can sign up at the ICLAS website at: https://iclas.org/genetic-monitoring-reference-program.

## Concluding remarks

Genetic Quality is a critical component that plays a major role in animal research but is often overlooked due to its complexity. We have addressed in this review the different genetic engineering technologies that are used in generating genetically modified mice and have pointed out ways to check them for genetic compliance. As part of ICLAS goals we are committed to assist the scientific community in establishing a genetic control program in their breeding facilities and help with its compliance so that scientific publications provide all the important elements of the strain and/or genetic modifications of the mouse model used. We hope that this review clarifies and brings awareness to the genetic quality of mouse model research.

## References

[CR1] Ackert-Bicknell CL, Rosen CJ (2016). Passenger gene mutations: unwanted guests in genetically modified mice. J Bone Miner Res.

[CR2] Babinet C, Cohen-Tannoudji M (2001). Genome engineering via homologous recombination in mouse embryonic stem (ES) cells: an amazingly versatile tool for the study of mammalian biology. An Acad Bras Ciênc.

[CR3] Bandrowski A, Brush M, Grethe JS et al (2015) The Resource identification initiative: a cultural shift in publishing [version 2; peer review: 2 approved]. F1000Research 4:134 10.12688/f1000research.6555.2 10.12688/f1000research.6555.1 PMC4648211 26594330

[CR4] Basta P, Whitmore S, Basham B, Whisnant C (2004). Microsatellite analysis in FVB/N mice. Comp Med.

[CR5] Becher B, Waisman A, Lu LF (2018). Conditional gene-targeting in mice: problems and solutions. Immunity.

[CR6] Beck JA, Lloyd S, Hafezparast M, Lennon-Pierce M, Eppig JT, Festing MF, Fisher EM (2000). Genealogies of mouse inbred strains. Nat Genet.

[CR7] Benavides F, Rülicke T, Prins J-B, Bussell J, Scavizzi F, Cinelli P (2020). Genetic quality assurance and genetic monitoring of laboratory mice and rats: FELASA Working Group Report. Lab Anim.

[CR8] Birling MC, Yoshiki A, Adams DJ (2021). A resource of targeted mutant mouse lines for 5,061 genes. Nat Genet.

[CR9] Boroviak K, Fu B, Yang F (2017). Revealing hidden complexities of genomic rearrangements generated with Cas9. Sci Rep.

[CR10] Bouabe H, Okkenhaug K (2013). Gene targeting in mice: a review. Methods Mol Biol.

[CR11] Bourdi M, Davies JS, Pohl LR (2011). Mispairing C57BL/6 substrains of genetically engineered mice and wild-type controls can lead to confounding results as it did in studies of jnk2 in acetaminophen and concanavalin a liver injury. Chem Res Toxicol.

[CR12] Browning J, Rooney M, Hams E (2020). Highly efficient CRISPR-targeting of the murine Hipp11 intergenic region supports inducible human transgene expression. Mol Biol Rep.

[CR13] Bryda EC, Riley LK (2008). Multiplex microsatellite marker panels for genetic monitoring of common rat strains. J Am Assoc Lab Anim Sci.

[CR14] Cain-Hom C, Splinter E, van Min M, Simonis M, van de Heijning M, Martinez M (2017). Efficient mapping of transgene integration sites and local structural changes in Cre transgenic mice using targeted locus amplification. Nucleic Acids Res.

[CR15] Church DM, Goodstadt L, Hiller LW, Zody MC, Goldstein S (2009). The Mouse genome sequencing consortium lineage specific biology revealed by a finished genome assembly of the mouse. PLoS Biol.

[CR16] Cohen J (2020). A cut above: pair that developed CRISPR earns historic award. Science.

[CR17] Coleman DL (1978). Obese and diabetes: two mutant genes causing diabetes-obesity syndromes in mice. Diabetologia.

[CR18] Colledge WH, Abella BS, Southern KW, Ratcliff R, Jiang C, Cheng SH, MacVinish LJ, Anderson JR, Cuthbert AW, Evans MJ (1995). Generation and characterization of a delta F508 cystic fibrosis mouse model. Nat Genet.

[CR19] Cong L, Ran FA, Cox D, Lin S, Barreto R, Habib N, Hsu PD, Wu X, Jiang W, Marrafini LA, Zhang F (2013). Multiplex genome engineering using CRISPR/Cas systems. Science.

[CR20] Cormack BP, Valdivia RH, Falkow S (1996). FACS-optimized mutants of the green fluorescent protein (GFP). Gene.

[CR21] Dallas JF (1992). Estimation of microsatellite mutation rates in recombinant inbred strains of mouse. Mamm Genome.

[CR22] DeChiara TM, Effstratiadis A, Robertson EJ (1990). A growth-deficiency phenotype in heterozygous mice carrying an insulin-like growth factor II gene disrupted by targeting. Nature.

[CR23] Doetschman T, Gregg RG, Maeda N, Hooper ML, Melton DW, Thompson S, Smithies O (1987). Targeted correction of a mutant HPRT gene in mouse embryonic stem cells. Nature.

[CR24] Dong Y, Li H, Zhao L, Koopman P, Zhang F, Huang JX (2019) Genome-wide Off-Target Analysis in CRISPR-Cas9 Modified Mice and Their Offspring. G3. 9:3645–3651 10.1534/g3.119.400503 10.1534/g3.119.400503 PMC6829146 31492696

[CR25] Duff K, Knight H, Refolo LM, Sanders S, Yu X, Picciano M, Malester B, Hutton M, Adamson J, Goedert M, Burki K, Davies P (2000). Characterization of pathology in transgenic mice over-expressing human genomic and cDNA tau transgenes. Neurobiol Dis.

[CR26] Eisener-Dorman AF, Lawrence DA, Bolivar VJ (2009). Cautionary insights on knockout mouse studies: The gene or not the gene?. Brain Behav Immun.

[CR28] Eppig JT (2006) Mouse strain and genetic nomenclature: an abbreviated guide. In: The mouse in biomedical research, Volume 1, Second Edition. Fox J, Barthold S, Davisson M, Newcomer C, Quimby F, Smith A, eds. Academic Press. pp 79–98

[CR27] Eppig JT, Motenko H, Richardson JE, Richards-Smith B, Smith CL (2015). The International Mouse Strain Resource (IMSR): cataloging worldwide mouse and ES cell line resources. Mamm Genome.

[CR29] Evans MJ, Kaufman MH (1981). Establishment in culture of pluripotential cells from mous. embryos. Nature.

[CR30] Fahey JR, Katoh H, Malcolm R, Perez AV (2013). The case for genetic monitoring of mice and rats used in biomedical research. Mamm Genome.

[CR31] Festing, MFW (1979) Inbreeding and its consequences, and the history of inbred strains, 3–20. In: Inbred strains in biomedical research, Macmillan Publishers Limited, London.

[CR32] Flurkey K, editor. The Jackson laboratory handbook of genetically standardized mice: ask for the j. 6. ed., 1. printing. Bar Harbor, Me: The Jackson Laboratory; 2009

[CR33] Fox, Richard R., Wiles, Michael V., Petkov, Petko M. Genetic Monitoring. 2nd ed. In: Fox JG, Davisson MT, Quimby FW, Barthold SW, Newcomer CE, Smith AL, editors. The Mouse in Biomedical Research. Second Edition. Burlington: Academic Press; 2007. pp. 321–326 10.1016/B978-012369454-6/50094-7

[CR34] Freedman LP, Cockburn IM, Simcoe TS (2015). The Economics of reproducibility in preclinical research. PLoS Biol.

[CR35] Freeman HC, Hugill A, Dear NT, Ashcroft FM, Cox RD (2006). Deletion of nicotinamide nucleotide transhydrogenase: a new quantitive trait locus accounting for glucose intolerance in C57BL/6J Mice. Diabetes.

[CR36] Green EL (1981). Genetics and probability in animal breeding experiments.

[CR37] Grover A, Sharma PC (2014). Development and use of molecular markers: past and present. Crit Rev Biotechnol.

[CR38] Hansen GM, Markesich DC, Burnett MB, Zhu Q, Dione KM, Richter LJ, Finnell RH, Sands AT, Zambrowicz BP, Abuin A (2008). Large scale gene trapping in C57BL/6N mouse embryonic stem cells. Genome Res.

[CR39] Heaney JD, Rettew AN, Bronson SK (2004). Tissue-specific expression of a BAC transgene targeted to the Hprt locus in mouse embryonic stem cells. Genomics.

[CR40] Ichise H, Ichise T, Sasanuma H, Yoshida N (2014). The Cd6 gene as a permissive locus for targeted transgenesis in the mouse. Genesis.

[CR41] Isola LM, Gordon JW (1991). Transgenic animals: a new era in developmental biology and medicine. Biotechnology.

[CR42] Ittner LM, Götz J (2007). Pronuclear injection for the production of transgenic mice. Nat Protoc.

[CR43] Iyer V, Boroviak K, Thomas M, Doe B, Riva L, Ryder E, Adams DJ (2018). No unexpected CRISPR-Cas9 off-target activity revealed by trio sequencing of gene-edited mice. PLoS Genet.

[CR44] Kim DW, Uetsuki T, Kaziro Y, Yamaguchi N, Sugano S (1990). Use of the human elongation factor 1 alpha promoter as a versatile and efficient expression system. Gene.

[CR45] Kosicki M, Tomberg K, Bradley A (2018). Repair of double-strand breaks induced by CRISPR-Cas9 leads to large deletions and complex rearrangements. Nat Biotechnol.

[CR46] Kraft K, Geuer S, Will AJ, Chan WL, Paliou C, Borschiwer M (2015). Deletions, inversions, duplications: engineering of structural variants using CRISPR/Cas in mice. Cell Rep.

[CR47] Kuno A, Ikeda Y, Ayabe S, Kato K, Sakamoto K, Suzuki SR (2022). DAJIN enables multiplex genotyping to simultaneously validate intended and unintended target genome editing outcomes. PLoS Biol.

[CR48] Lamb BT, Bardel KA, Kulnane LS, Anderson JJ, Holtz G, Wagner SL, Sisodia SS, Hoeger EJ (1999). Amyloid production and deposition in mutant amyloid precursor protein and presenilin-1 yeast artificial chromosome transgenic mice. Nat Neurosci.

[CR49] Leenaars CHC, Kouwenaar C, Stafleu FR (2019). Animal to human translation: a systematic scoping review of reported concordance rates. J Transl Med.

[CR50] Lilue J, Doran AG, Fiddes IT, Abrudan M, Armstrong J, Bennett R (2018). Sixteen diverse laboratory mouse reference genomes define strain-specific haplotypes and novel functional loci. Nat Genet.

[CR51] Little CC, Tyzzer EE (1916). Further experimental studies on the inheritance of susceptibility to a Transplantable tumor, Carcinoma (J. W. A.) of the Japanese waltzing Mouse. J Med Res.

[CR52] Lloyd K, Franklin C, Lutz C, Magnuson T (2015). Reproducibility: use mouse biobanks or lose them. Nature.

[CR53] Lutz CM, Linder CC, Davisson MT, Hedrich HJ (2012). Strains, Stocks and Mutant Mice. The Laboratory Mouse.

[CR54] Ma Y, Yu L, Pan S, Gao S, Chen W, Zhang X, Dong W, Li J, Zhou R, Huang L, Han Y, Bai L, Zhang L, Zhang L (2017). CRISPR/Cas9-mediated targeting of the Rosa26 locus produces Cre reporter rat strains for monitoring Cre-loxP-mediated lineage tracing. FEBS J.

[CR55] Madisen L, Garner AR, Shimaoka D, Chuong AS, Klapoetke NC, Li L, van der Bourg A, Niino Y, Egolf L, Monetti C, Gu H, Mills M, Cheng A, Tasic B, Nguyen TN, Sunkin SM, Benucci A, Nagy A, Miyawaki A, Helmchen F, Empson RM, Knopfel T, Boyden ES, Reid RC, Carandini M, Zeng H (2015). Transgenic mice for intersectional targeting of neural sensors and effectors with high specificity and performance. Neuron.

[CR56] Mahler M, Nicklas W, Hedrich HJ (2012). Genetic Monitoring of Inbred Strains of Mice. Second. The Laboratory Mouse.

[CR57] Mansour SL, Thomas KR, Capecchi MR (1988). Disruption of the proto-oncogene int-2 in mouse embryo-derived stem cells: a general strategy for targeting mutations to non-selectable genes. Nature.

[CR58] Mattapallil MJ, Wawrousek EF, Chan C-C, Zhao H, Roychoudhury J, Ferguson TA (2012). The *Rd8* mutation of the *Crb1* Gene Is present in vendor lines of C57BL/6N mice and embryonic stem cells, and confounds ocular induced mutant phenotypes. Invest Ophthalmol vis Sci.

[CR59] Meek S, Mashimo T, Burdon T (2017). From engineering to editing the rat genome. Mamm Genome.

[CR60] Mekada K, Yoshiki A (2021). Substrains matter in phenotyping of C57BL/6 mice. Exp Anim.

[CR61] Mekada K, Hirose M, Murakami A, Yoshiki A (2015). Development of SNP markers for C57BL/6N-derived mouse inbred strains. Exp Anim.

[CR62] Mizuno S, Dinh TT, Kato K, Mizuno-Iijima S, Tanimoto Y, Daitoku Y (2014). Simple generation of albino C57BL/6J mice with G291T mutation in the tyrosinase gene by the CRISPR/Cas9 system. Mamm Genome.

[CR63] Morgan AP, Fu C-P, Kao C-Y, Welsh CE, Didion JP, Yadgary L (2016). The mouse universal genotyping array: from substrains to subspecies. G3.

[CR64] Morse, H. C. III (2007) Breeding a better mouse: one hundred years of genetics and biology, 1–11. In: history, wild mice, and genetics. The mouse in biomedical research. Edited by J.G. Fox, S.W. Barthold, M.T. Davisson, C.E. Newcomer, F.W. Quimby and A.L. Smith. Second edition. Elsevier, Inc., London.

[CR65] Mouse Genome Sequencing Consortium (2002). Initial sequencing and comparative analysis of the mouse genome. Nature.

[CR66] MPD: Mouse Phenome Database: Welcome. [cited 2 Dec 2020]. Available: https://phenome.jax.org/

[CR67] Myakishev MV (2001). High-throughput SNP genotyping by allele-specific pcr with universal energy-transfer-labeled primers. Genome Res.

[CR68] Nagai T, Ibata K, Park ES, Kubota M, Mikoshiba K, Miyawaki A (2002). A variant of yellow fluorescent protein with fast and efficient maturation for cell-biological applications. Nat Biotechnol.

[CR69] Nagy A (2000). Cre recombinase: the universal reagent for genome tailoring. Genesis.

[CR70] Nagy A, Gertsenstein M, Vintersten K, Behringer R (2003) Vector design for embryonic stem cell-based transgenesis and genome alterations. pp399–429. In Manipulating the mouse embryos: a laboratory manual. The 3rd Edition. Cold Spring Harbor Laboratory Press.

[CR71] Nakashiba T, Young JZ, McHugh TJ, Buhl DL, Tonegawa S (2008). Transgenic inhibition of synaptic transmission reveals role of CA3 output in hippocampal learning. Science.

[CR72] Nakata H, Hashimoto T, Seki Y, Mekada K, Obata Y, Yoshiki A (2009). Simultaneous detection of multiple transgenes for genetically-modified mouse strains. Exp Anim.

[CR73] Nakata H, Hashimoto T, Yoshiki A (2021). Quick validation of genetic quality for conditional alleles in mice. Genes Cells.

[CR74] Ohtsuka M, Miura H, Mochida K, Hirose M, Hasegawa A, Ogura A, Mizutani R, Kimura M, Isotani A, Ikawa M, Sato M, Gurumurthy CB (2015). One step generation of multiple transgenic mouse lines using an improved pronuclear injection-based targeted transgenesis *i*-PITT. Genomics.

[CR75] Paigen K (2003). One hundred years of mouse genetics: an intellectual history. I. the classical period (1902–1980). Genetics.

[CR76] Palais G, Nguyen Dinh Cat A, Friedman H, Panek-Huet N, Millet A, Tronche F, Gellen B, Mercadier JJ, Peterson A, Jaisser F (2009). Targeted transgenesis at the HPRT locus: an efficient strategy to achieve tightly controlled in vivo conditional expression with the tet system. Physiol Genomics.

[CR77] Palmiter RD, Brinster RL (1985). Transgenic mice. Cell.

[CR78] Palmiter RD, Brinster RL (1986). Germ-line transformation of mice. Annu Rev Genet.

[CR79] Percie du Sert N, Hurst V, Ahluwalia A, Alam S, Avey MT, Baker M (2020). The ARRIVE guidelines 2.0: Updated guidelines for reporting animal research. PLoS Biol.

[CR80] Petkov PM, Cassell MA, Sargent EE, Donnelly CJ, Robinson P, Crew V (2004). Development of a SNP genotyping panel for genetic monitoring of the laboratory mouse. Genomics.

[CR81] Pettitt SJ, Liang Q, Rairdan XY, Moran JL, Prosser HM, Beier DR, Lloyd KC, Bradley A, Skarnes WC (2009). Agouti C57BL/6N embryonic stem cells for mouse genetic resources. Nat Methods.

[CR82] Polites HG, Pinkert CA (1994) DNA microinjection and transgenic animal production. pp. 15–68. In Transgenic Animal Technology, Laboratory Handbook. Edited by Pinkert CA. Academic Press, Inc.

[CR83] Poltorak A, He X, Smirnova I, Liu MY, Van Huffel C, Du X (1998). Defective LPS signaling in C3H/HeJ and C57BL/10ScCr mice: mutations in Tlr4 gene. Science.

[CR84] Potter, M (1985) History of the BALB/c family. pp 1–5. In Current Topics in Microbiology and Immunology 122: The BALB/c Mouse - Genetics and Immunology (M. Potter, Ed.)

[CR85] Rogner UC, Avner P (2003). Congenic mice: cutting tools for complex immune disorders. Nat Rev Immunol.

[CR86] Roguev A, Krogan NJ (2008). BAC to the future: functional genomics in mammals. Nat Methods.

[CR87] Sadowski PD (1995). The Flp recombinase of the 2-microns plasmid of Saccharomyces cerevisiae. Prog Nucleic Acid Res Mol Biol.

[CR88] Sauer B, Henderson N (1988). Site-specific DNA recombination in mammalian cells by the Cre recombinase of bacteriophage P1. Proc Natl Acad Sci U S A.

[CR89] Schönig K, Freundlieb S, Gossen M (2013). Tet-Transgenic Rodents: a comprehensive, up-to date database. Transgenic Res.

[CR90] Sigmon JS, Blanchard MW, Baric RS, Bell TA, Brennan J, Brockman GA (2020). Content and performance of the MiniMUGA genotyping array: a new tool to improve rigor and reproducibility in mouse research. Genetics.

[CR91] Simon MM, Greenaway S, White JK, Fuchs H, Gailus-Durner V, Wells S (2013). A comparative phenotypic and genomic analysis of C57BL/6J and C57BL/6N mouse strains. Genome Biol.

[CR92] Skarnes W, Rosen B, West A (2011). A conditional knockout resource for the genome-wide study of mouse gene function. Nature.

[CR93] Smirnov A & Battulin N. (2021) Concatenation of transgenic DNA: Random or orchestrated. Genes 12:1969 10.3390/genes12121969 10.3390/genes12121969 PMC8701086 34946918

[CR94] Smithies O, Gregg RG, Boggs SS, Doralewski MA, Kucherlapati RS (1985). Insertion of DNA sequences into the human chromosomal beta-globin locus by homologous recombination. Nature.

[CR95] Soriano P (1999). Generalized lacZ expression with the ROSA26 Cre reporter strain. Nat Genet.

[CR96] Strobel MC, Reinholdt LG, Malcolm RD, Pritchett-Corning K (2015) Chapter 31 - Genetic Monitoring of Laboratory Mice and Rats. In: Fox JG, Anderson LC, Otto GM, Pritchett-Corning KR, Whary MT, editors. Laboratory Animal Medicine (Third Edition). Boston: Academic Press pp. 1403–1416 10.1016/B978-0-12-409527-4.00031-6

[CR97] Tanimoto Y, Iijima S, Hasegawa Y, Suzuki Y, Daitoku Y, Mizuno S, Ishige T, Kudo T, Takahashi S, Kunita S, Sugiyama F, Yagami K (2008). Comp Med.

[CR98] Tasic B, Hippenmeyer S, Wang C, Gamboa M, Zong H, Chen-Tsai Y, Luo L (2011). Site-specific integrase-mediated transgenesis in mice via pronuclear injection. Proc Natl Acad Sci USA.

[CR99] Thomas KR, Capecchi MR (1987). Site-directed mutagenesis by gene targeting in mouse embryo-derived stem cells. Cell.

[CR100] Threadgill D, Dlugosz A, Hansen L, Tennenbaum T, Lichti U, Yee D (1995). Targeted disruption of mouse EGF receptor: effect of genetic background on mutant phenotype. Science.

[CR101] Tinkle BT, Bieberich CJ, Jay G (1994) Molecular approaches involved in mammalian gene transfer: analysis of transgene integration. pp. 221–234. In Transgenic Animal Technology, Laboratory Handbook. Edited by Pinkert CA. Academic Press, Inc.

[CR102] Tsien RY (1998). The green fluorescent protein. Annu Rev Biochem.

[CR103] Turner PV, Pekow C, MacArthur Clark J, Vergara P, Bayne K, White WJ (2015). Roles of the International Council for Laboratory Animal Science (ICLAS) and International Association of Colleges of Laboratory Animal Medicine (IACLAM) in the Global Organization and Support of 3Rs Advances in Laboratory Animal Science. J Am Assoc Lab Anim Sci.

[CR104] Vasilevsky NA, Brush MH, Paddock H, Ponting L, Tripathy SJ, LaRocca GM, Haendel MA (2013). On the reproducibility of science: unique identification of research resources in the biomedical literature. PeerJ.

[CR105] Vivian JL, Klein WH, Hasty P (1999). Temporal, spatial and tissue-specific expression of a myogenin-lacZ transgene targeted to the Hprt locus in mice. Biotechniques.

[CR106] Vogel G (2007). A knockout award in medicine. Science.

[CR107] Wakeland E, Morel L, Achey K, Yui M, Longmate J (1997). Speed congenics: a classic technique in the fast lane (relatively speaking). Immunol Today.

[CR108] Wang H, Yang H, Shivalila CS, Dawlaty MM, Cheng AW, Zhang F, Jaenisch R (2013). One-step generation of mice carrying mutations in multiple genes by CRISPR/Cas-mediated genome engineering. Cell.

[CR109] Yang XW, Model P, Heintz N (1997). Homologous recombination based modification in Escherichia coli and germline transmission in transgenic mice of a bacterial artificial chromosome. Nat Biotechnol.

[CR110] Yang TT, Sinai P, Green G, Kitts PA, Chen YT, Lybarger L (1998). Improved fluorescence and dual color detection with enhanced blue and green variants of the green fluorescent protein. J Biol Chem.

[CR111] Yang H, Wang H, Jaenisch R (2014). Generating genetically modified mice using CRISPR/Cas-mediated genome engineering. Nat Protoc.

[CR112] Yeh CD, Richardson CD, Corn JE (2019). Advances in genome editing through control of DNA repair pathways. Nat Cell Biol.

[CR113] Zeng H, Horie K, Madisen L, Pavlova MN, Gragerova G, Rohde AD, Schimpf BA, Liang Y, Ojala E, Kramer F, Roth P, Slobodskaya O, Dolka I, Southon EA, Tessarollo L, Bornfeldt KE, Gragerov A, Pavlakis GN, Gaitanaris GA (2008). An inducible and reversible mouse genetic rescue system. PLoS Genet.

[CR114] Zevnik B, Uyttersprot NC, Perez AV, Bothe GWM, Kern H, Kauselmann G (2014). C57BL/6N Albino/Agouti mutant mice as embryo donors for efficient germline transmission of C57BL/6 ES cells. PLoS ONE.

